# Novel quantum description for nonadiabatic evolution of light wave propagation in time-dependent linear media

**DOI:** 10.1038/srep19860

**Published:** 2016-02-05

**Authors:** Halim Lakehal, Mustapha Maamache, Jeong Ryeol Choi

**Affiliations:** 1 Laboratoire de Physique Quantique et Systèmes Dynamiques, Faculté des Sciences, Université Ferhat Abbas Sétif 1, Sétif 19000, Algeria; 2 Department of Radiologic Technology, Daegu Health College, Buk-gu, Daegu 41453, Republic of Korea

## Abstract

A simple elegant expression of nonadiabatic light wave evolution is necessary in order to have a deeper insight for complicated optical phenomena in light science as well as in everyday life. Light wave propagation in linear media which have time-dependent electromagnetic parameters is investigated by utilizing a quadratic invariant of the system. The time behavior of the nonadiabatic geometric phase of the waves that yield a cyclic nonadiabatic evolution is analyzed in detail. Various quantum properties of light waves in this situation, such as variances of electric and magnetic fields, uncertainty product, coherent and squeezed states, and their classical limits, are developed. For better understanding of our research, we applied our analysis in a particular case. The variances of the fields **D** and **B** are illustrated and their time behaviors are addressed. Equivalent results for the corresponding classical systems are deduced from the study of the time evolution of the appropriate coherent and squeezed states.

If electromagnetic parameters of the media, such as permittivity, magnetic permeability, and conductivity, change as time goes by, the media are classified as time-dependent media. Recently, great attention has been paid to achieving the accurate and efficient description for light wave propagation in time-dependent linear and random media; this is partly due to the concern to quantum optics processes in modern optical materials such as optical fibers^1^,^2^,^3^,^4^,^5^,^6^,^7^,^8^,^9^. The light waves in such media are described by a time-dependent Hamiltonian on account of the time-dependence of parameters. It is interesting to note that, even if the time-dependence of parameters disappears, the Hamiltonian of an electromagnetic field is time-dependent as long as the conductivity exists^7^,^10^.

There exist generally accepted approaches for developing the quantum theory of light waves of which the corresponding Hamiltonian is explicitly time-dependent. Some of us^8^,^9^, studied the geometric phase of quantized light waves in time-dependent linear media, where the parameters of the system undergo adiabatic change. They considered the eigenstates of a time-dependent Hamiltonian in order to derive the adiabatic geometric phase change under the assumption that the parameters of the system vary sufficiently slowly with time. Their research is principally based on Berry’s report appearing in his classic paper^11^, which states that the wave function acquires a geometric phase (now known as Berry’s phase) in addition to the usual dynamical phase when a physical system evolves in a cyclic and adiabatic fashion.

In contrast to the adiabatic process of phase change treated in the previous works^8^,^9^, we, in this paper, investigate the dynamical properties of nonadiabatic geometric phase accumulated by a somewhat fast change of control parameters of the medium for a light wave propagating in time-dependent linear media. For this reason, we introduce a quadratic invariant of Lewis and Riesenfeld^12^ and consider the eigenstates of this invariant (instead of those of the Hamiltonian) in order to study the features of the geometric phase. Then the quantum description of electromagnetic field whose Hamiltonian is time-dependent can be achieved on a fundamental level by taking advantage of the invariant formulation of quantum electrodynamics.

The importance of Berry’s finding of the geometric phase and its impact on various areas of physics have naturally led to arousing the interest in the generalizations of geometric phases. One of the most significant contributions in this direction is the nonadiabatic generalization of Berry’s phase firstly fulfilled by Aharonov and Anandan^13^. This generalization employs a geometric picture of quantum dynamics and shows that the nonadiabatic geometric phase can be defined for any closed curve in the space of (pure) quantum states. Perhaps one of the most important research studies connected to these is the classic seminal paper of Lewis and Riesenfeld^12^ on dynamical invariants. The correspondence of Berry’s phase and Lewis’s phase has been pointed out by Morales^14^. The classical counterpart of Aharonov-Anandan quantum geometric phase is the nonadiabatic Hannay angle^15^,^16^. Since the invariant action proposed by Lewis^12^ exists independently of whether the Hamiltonian is changed slowly or not, the geometric angle can be defined on constant-action tori for a cyclic evolution, independently of whether the evolution is adiabatic or not. The appropriate interpretation of the angle obtained in this way is the classical counterpart (Hannay angle) of the geometric phase of the Aharonov and Anandan^13^. For light waves described by the generalized harmonic oscillator, the adiabatic-approximation-results can be obtained from an asymptotic theory of nonadiabatic process.

To investigate the problem of the nonadiabatic geometric phase for quantized light waves propagating through homogeneous conducting linear media, we use Coulomb’s gauge and assume that the medium has no free charge for simplicity. We reduce the problem to that of a generalized time-dependent harmonic oscillator Hamiltonian and we examine the geometric character of light waves with the help of the dynamical invariant. From Maxwell’s equations, we obtain the classical Hamiltonian for the light waves propagating through homogeneous conducting linear media without charge density and we survey the basic results of the dynamical invariants and their relationship with geometric angles. In particular, the expression of electromagnetic fields in terms of the nonadiabatic geometric angle will be derived. We will address the characteristics of the geometrically equivalent quantum systems and, through a construction of annihilation and creation operators, we will investigate the quantum properties of light in time-dependent linear media. The coherent and squeezed states of the generally described light wave will be investigated and the adiabatic limit of our results will be compared to the previously known ones.

## Materials and Methods

In this section, we investigate the nonadiabatic geometric phase of light waves in time-dependent linear media using invariant theory developed for time-dependent Hamiltonian systems. Since the Hamiltonian is nonconservative in this situation, the invariant related approach for the geometric phase is useful now.

In time-dependent linear media, the relations between fields and current are given by


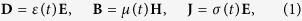


where 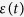, 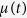, and 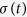 are time-dependent electric permittivity, magnetic permeability, and conductivity, respectively. Due to the time-dependence of the parameters, the speed of a light wave varies with time and is given by 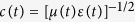. In the Coulomb gauge, Maxwell’s equations in time-dependent linear media give a damped wave equation for the vector potential such that^4^,^8^ 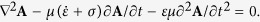 To separate 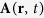 into mode function 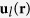 and time function 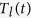, we put


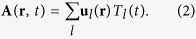


Then, 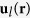 satisfies 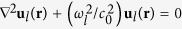, while 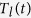 can be written as^8^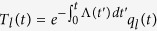 with 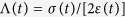, and 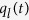 yields


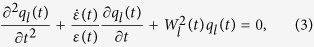


where 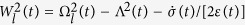, 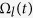 is a time-dependent natural frequency, 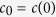, and 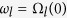. From the fact that the wave number 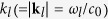 does not vary with time, we have 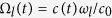.

The mode function is determined by geometry and boundary conditions. For instance, the mode function for a light wave traveling under periodic boundary condition is given by 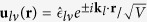 where *V* is the volume of a sector and 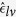 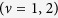 are unit vectors that indicate the direction of the polarization for the electromagnetic field.

Considering Eq. (3), we can easily show from Hamilton’s equations that 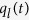 is described by a classical generalized harmonic oscillator (GHO) Hamiltonian of the form^8^


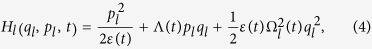


where 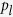 is the canonical conjugate variable of 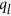. By summing all of the Hamiltonian associated to each mode function, we have the total Hamiltonian 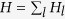. Because the scalar potential disappears in charge free space, the electric and the magnetic fields are represented only in terms of the vector potential such that


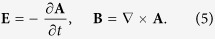


Using the first relation in the above equation along with Eqs. (2) and (4), we see that the electric field can be represented in the form


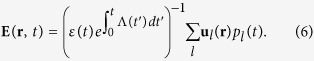


Let us recall that the general method to introduce geometric angles related to invariants is valid regardless of whatever form of the time dependence for the parameters^16^. For a system specified by a time-dependent Hamiltonian 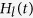, a nontrivial invariant 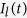 obeys the equation 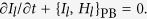 A remarkable property of this Hamiltonian system is that any initial tori in phase space, which are surfaces of constant action 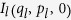 parameterized by the angle 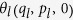 at initial time 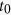, evolve into the tori identified with constant-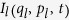 surfaces, according to a flow produced by the Hamiltonian 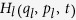. 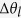 is the angle by which any phase point on the torus is shifted at time *t* from its value at 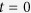.

It follows from the Hamilton’s equations that the rate of change of the angle of a phase point is determined from the sum of contributions produced by its motion in phase space and by the changing coordinates 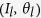:


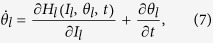


where 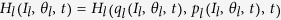. By integrating Eq. (7), we obtain 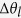, which does not depend on 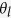; however, the sum in each term does depend on 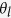. These dependences can be eliminated by averaging over each contour of constant action, thus 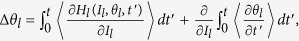 where 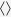 denotes the average over 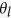 at a fixed time. The first term is the dynamical angle 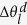 and the second is the classical geometric angle 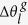.

For the GHO, exact calculations of a Hamiltonian-like quadratic invariant can be made and the explicit form of the resultant invariant and of the associated angle are found, for example, in refs 16, 17, 18, 19, 20. Such invariant is read as


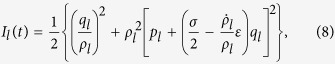


where 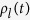 is a c-number solution of the auxiliary equation


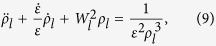


and performing some algebra for the angle with Eq. (7) leads to


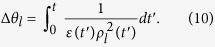


Then, the exact solution for 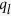 is given by


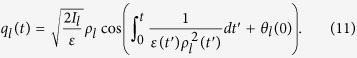


This oscillates in time with a time-dependent phase increment.

## Results

### Effects of Geometric Phase on Radiation Fields

Now let us see the effects of the nonadiabatic geometric phase for the light wave propagating under a periodic boundary condition. If we replace the classical variables 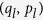 with operators 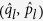 where 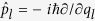, it is possible to investigate this problem in view of quantum mechanics. Each mode of the electromagnetic field behaves like a time-dependent harmonic oscillator whose quantum features are clearly understood through the introduction of annihilation and creation operators associated with the invariant presented in the previous section. If we consider an annihilation operator and a creation operator that are given by


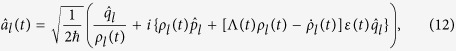



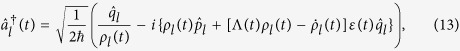


the invariant, Eq. (8), reduces to a simple form:


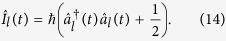


One can easily verify that 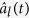 and 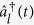 satisfy the boson commutation relation 
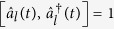
. From the result of the direct differentiation of 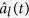 with respect to time:


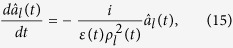


we see that the time evolution of the annihilation operator is given by


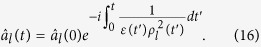


In terms of the canonical variable


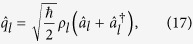


obtained from the inverse representation of 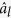 and 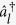 (given in Eqs. (12) and (13)), the vector potential given in Eq. (2) can be completely described as


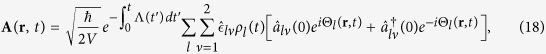


for the traveling light waves, where


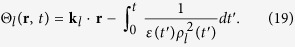


Then, from the basic relations given in Eq. (5) and the first term of Eq. (1), we see that the electric displacement and the magnetic field operators are represented in the form


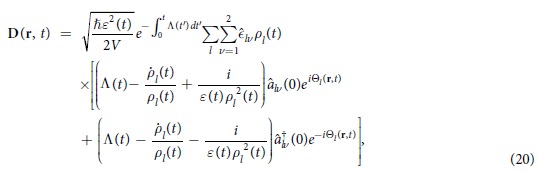



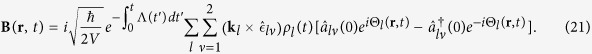


Due to the existence of the exponential factor 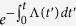 in these equations, both the electric and the magnetic fields decrease with time according to the electromagnetic energy dissipation produced by the conductivity 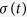 in media. We can confirm from this that the time behavior of the amplitude of the fields are the same as that of the classical fields. Of course, similar behavior appears in the case of adiabatic change of geometric phase^8^,^9^. In the limit 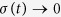, the fields no longer dissipate with time as expected.

We are able to understand the geometric character of the light wave from the phase factor 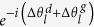. As you can see, the light waves undergo geometric phase change 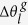 as well as familiar change of dynamical phase, 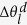. The interference fringe, produced when two or more light waves with different modes meet from different paths, would be altered more or less by the existence of the geometric phase. This concept is important for accurate prediction of interference pattern in interferometers.

### Adiabatic Limit: Phase Splitting into Dynamical and Geometrical Parts

One can find the adiabatic limit of the expressions presented up to now by using the following argument. In the adiabatic regime, the differentiation of 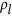 with respect to time induces a multiplication by a small adiabatic parameter 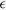, i.e., 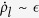. Thus, concerning the expression of the invariant given in Eq. (8), one can neglect the term involving 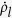 and replace 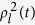 in the remaining ones by the zeroth-order solution of Eq. (9) with respect to 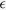; that is, 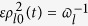 where 
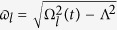
. One finds the adiabatic invariant of the generalized harmonic oscillator (GHO):


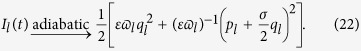


In the same way, for the phase, one can replace 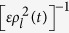 on the rhs of Eq. (10) by the approximate solution of Eq. (9), 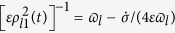, which is valid up to the first order in 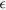; one then recovers the well known relation^8^


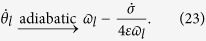


The time derivative of the angle, 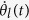, consists of two parts. The first one, 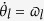, which exists even for a system with fixed parameters, corresponds to the so-called dynamical component of the angle, while the second one, 
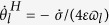
 is the (time derivative of the) geometrical Hannay’s phase.

For dielectric materials 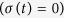, the geometric phase vanishes. Therefore, we see that the geometric properties of **E** and **B** fields vary depending on the characteristics of the medium. Moreover, the geometric phase appears when the medium becomes a conducting one with a slowly time-dependent conductivity while the electric permittivity is finite.

### Classical Limit: Coherent and Squeezed States for the Generalized GHO Model

The classical counterparts of the results developed up to now, i.e., the identity of the phase properties for the classical GHO and the expression of the classical GHO invariant (which is a generalized one of the results of refs 8,9 established under the restrictive adiabatic hypothesis), can be derived from classical limit of the quantum analysis. Indeed, the classical invariant and angle variables can be obtained by utilizing the quantum evolution of coherent states and squeezed states^21^,^22^. These states play the same role as the ordinary coherent states in the harmonic oscillator when considering the quantum-classical correspondence^23^,^24^.

The coherent state is an eigenstate of the annihilation operator 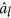:


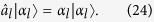


We can confirm from Eq. (16) that the eigenvalue is given by


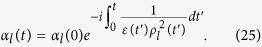


In some cases, it may be useful to recast the initial eigenstate in the form 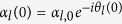 where 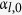 and 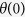 are real. To study these states for the GHO, recall Eq. (17) and consider the expression


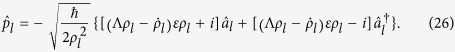


Then, by taking into account Eqs. (17) and (24, 25, 26), the expectation values of the canonical variables in the coherent state are given by


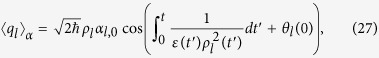



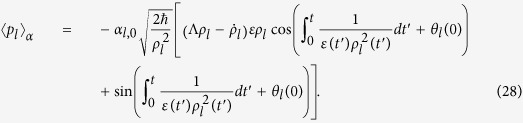


These quantities oscillate like those in the classical states. Indeed, coherent state is very much the same as classical state so far as the quantum mechanics allows. Like the quantum case, the phase of oscillation is governed by the time-dependent factor 
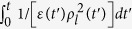
 that apparently involves the nonadiabatic part of the geometric phase.

The variances of 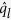 and 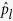 are


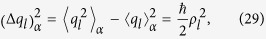



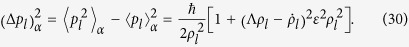


By multiplying the above two equations, we have the corresponding uncertainty product as


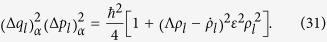


Now let us see the squeezed state. For this purpose, it is necessary to introduce an operator 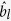 which is


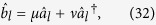


where *μ* and 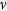 are complex parameters that follow 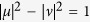. We can easily check that 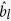 and its hermitian adjoint 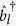 satisfy 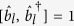. If we represent the eigenvalue equation for 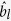 in the form


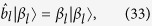


then 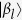 is the squeezed state. In general, the squeezed state is obtained by first squeezing the vacuum state and then displacing it^25^. The expectation value of canonical variables in the squeezed state, 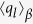 and 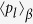, are identical to those in the coherent state given in Eqs. (27) and (28). Hence, the corresponding characteristics of the geometric phase for the canonical variables are very much the same as those of the coherent state.

However, the variances are different and their straightforward evaluations yield


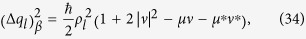



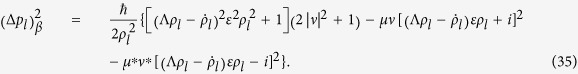


It is well known that one can make the size of 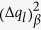 sufficiently small at the expense of a relative increase of the value 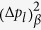 or vice versa. Consequently, the uncertainty product is


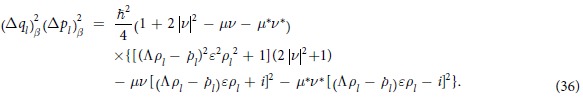


For *μ* = 1 and *v* = 0, the above equation reduces to that of the coherent state presented in Eq. (31).

### Behavior of Fields in the Coherent State

It is very interesting to analyze the expectation values and variances of the field operators in the coherent state. Using Eqs. (18)–(21), 77, 78, 79, we derive the expectation values of *l*th-mode field operators:


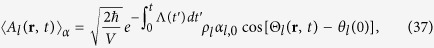



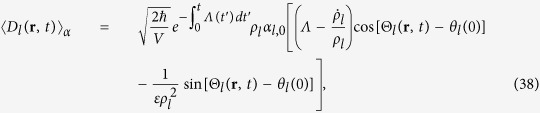



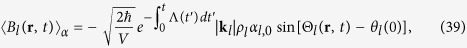


Notice that these expectation values in the coherent state oscillate sinusoidally according to the increment of phase angle 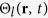. The amplitudes of such oscillations decrease with time on account of the presence of the time-dependent phase 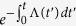.

Further, we can derive the variances of the field operators by means of methods similar to those of the previous evaluations for canonical variables:


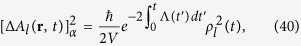



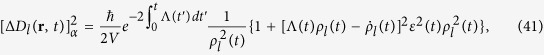



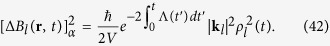


Considering the exponential term 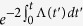, we can see that these variances also decrease with time.

For better understanding of the characteristics of light wave propagations developed here, let us consider for a particular case that 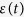 is given by


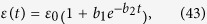


where 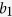 and 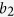 are real constants with the condition that 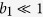. We further assume that *μ* and *σ* are real constants: 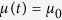 and 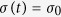. Then, it is possible to approximate


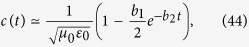



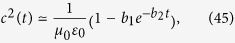



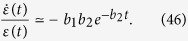


In this case, the equation for 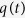 given in Eq. (3) becomes


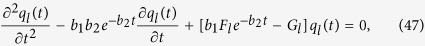


where


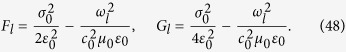


For convenience we consider only the case where 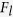 and 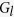 are positive which corresponds to the case 
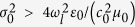
. Then, through the transformation 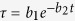, Eq. (47) becomes


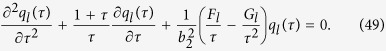


If we consider long time behavior 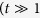, 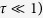, we easily have 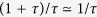. Then, the solution of Eq. (49) in this approximation is given by


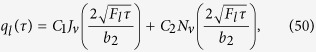


where 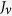 and 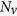 are Bessel functions of the first and the second kinds, respectively, while 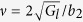. This equation can be rewritten in terms of *t* as


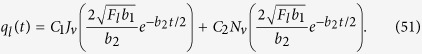


Although we have considered the long time behavior of the system in this approximated evaluation, the solution Eq. (51) can also be a good result for any initial time because of the obvious relation 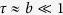 which is generally valid for small *t*.

The solution of Eq. (9) can be represented in terms of the two linearly independent solutions of Eq. (3), i.e., in terms of 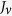 and 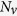 given in Eq. (51) 26, such that


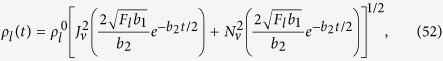


where 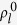 is a constant.

**Figure 1:**
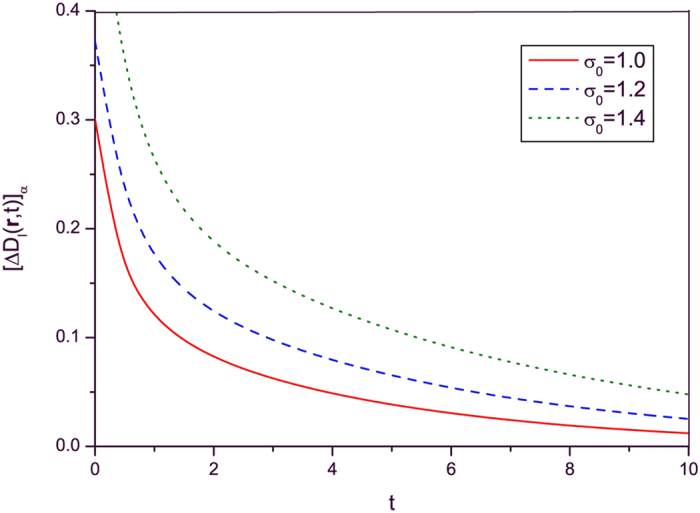
Time evolution of [Δ*D*_*l*_(r, *t*)]_*α*_ given in Eq. (41) where *ε*(*t*) is given by Eq. (43) and *μ* and *σ* are constants [*μ*(*t*) = *μ*_0_, *σ*(*t*) = *σ*_0_]. Several values of *σ*_0_ are taken as indicated in the figure. All other values are common and given by 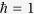, 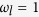, 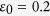, 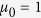, 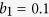, 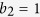, 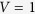, and 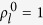. All values are taken to be dimensionless for the sake of convenience. This convention will also be used in the subsequent figure.

**Figure 2:**
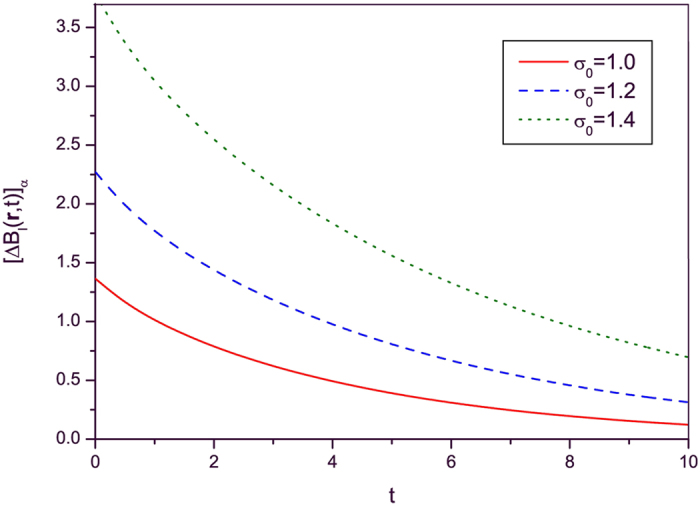
Time evolution of [Δ*B*_*l*_(r, *t*)]_*α*_ given in Eq. (42) with the same choice of time functions *ε*(*t*), *μ*(*t*) and *σ*(*t*) as those of Fig. 1 for several different values of *σ*_0_. The values of 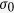 for each graph are the same as those of Fig. 1. All other values and conventions are also identical to those of Fig. 1.

The time evolutions of 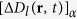 and 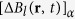 given in Eqs. (41) and (42) are plotted in Figs. 1 and 2 respectively for this particular case. When we drew these figures, the following integral relation is used for evaluating detailed numerical results:


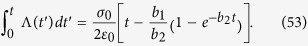


From these figures, we see that both 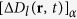 and 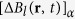 decrease with time as previously predicted. The amplitude of electric and magnetic fields and their variances gradually disappear because the conductivity plays the role of a dissipation factor.

## Discussion

Starting from the basic Maxwell’s equations, the GHO model of quantized light wave in time-dependent linear media was investigated with emphasis on its nonadiabatic evolution and accompanying properties of geometric phase. For convenience, we have considered charge free space and took advantage of the Coulomb gauge. Various quantum properties associated with light wave propagation in such situations and their adiabatic and classical limits were analyzed.

The vector potential 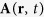 was separated into position function 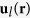 and time function 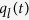 as shown in Eq. (2). In terms of 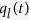 and its conjugate variable 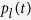, the time-dependent Hamiltonian of the system was constructed. To derive quantum solutions of the system, a Hamiltonian-like quadratic invariant was introduced. We can see from Eq. (14) that this invariant is represented as a simple form in terms of the annihilation and the creation operators. If we consider Eq. (16), the absolute value of the annihilation operator does not vary with time even if 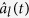 is an explicit function of time. With the use of the annihilation and the creation operators, full expressions of the field operators 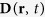 and 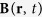 are derived and presented in Eqs. (20) and (21), respectively.

The expectation values of 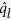 and 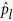 and their variances in the coherent and the squeezed states have been investigated. In the limit 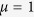 and 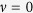, the corresponding uncertainty product in the squeezed state reduces to that of the coherent state. The expectation values of the field operators 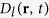 and 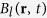 have also been derived. We have confirmed that such expectation values in the coherent state oscillate sinusoidally according to the increment of phase angle 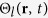. This time behavior is exactly the same as that of the classical fields. Not only the field operators but also their variances decrease with time due to the existence of the conductivity. This analysis agrees with the characteristics of classical fields.

We have studied the effects of time-varying parameters of the medium in nonadiabatic evolution of the geometric phase of a light wave. The geometric phase exhibits gauge structure relevant to a phase shift in nonadiabatic processes. There appears a classical analogue of this phase, which is the Hannay angle that can be formulated using the theory of action variable in the canonical structure of light wave phenomena. The adiabatic limit of the phase factor was investigated and it is shown in this limit that the angle 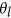 exactly recovers Eq. (23), where the first term is an ordinary dynamical component of the angle and the second one is the geometrical Hannay angle. We can see from the second term that the geometric phase change takes place when the conductivity varies slowly with time.

Finally, we note that there are several potential scientific applications of the results of this work, relevant to nonadiabatic geometric phase. One is a technique for quantum computation that can be carried out by using superconducting nanocircuits^27^ or nuclear magnetic resonance (NMR)^28^. For instance, a method to implement the Deutsch-Jozsa algorithm and Grover’s search algorithm using the nonadiabatic geometric phase in a two-qubit system has been suggested^28^. Our results can also be applied to the problem of shortcuts to adiabaticity^29^ that are introduced for speeding up quantum adiabatic processes. If we consider the fact that adiabatic processes are ubiquitous, the theory of shortcuts to adiabaticity can be employed to various fields in physics relevant to dynamical systems, ranging from the population inversion in two-level quantum systems^30^ to the trapping and control of Bose gases^31^. Another branch applicable to this work is to investigate the characteristics of quantized light waves in oscillating turbulent plasma, which is important for diagnosing and controlling the plasma state in a tokamak where nuclear fusion takes place^32^.

## Additional Information

**How to cite this article**: Lakehal, H. *et al.* Novel quantum description for nonadiabatic evolution of light wave propagation in time-dependent linear media. *Sci. Rep.* **6**, 19860; doi: 10.1038/srep19860 (2016).
